# Inference of chromosome-specific copy numbers using population haplotypes

**DOI:** 10.1186/1471-2105-12-194

**Published:** 2011-05-24

**Authors:** Yao-Ting Huang, Min-Han Wu

**Affiliations:** 1 Department of Computer Science and Information Engineering, National Chung Cheng University, Chia-Yi 621, Taiwan

## Abstract

**Background:**

Using microarray and sequencing platforms, a large number of copy number variations (CNVs) have been identified in humans. In practice, because our human genome is a diploid, these platforms are limited to or more accurate for detecting total copy numbers rather than chromosome-specific copy numbers at each of the two homologous chromosomes. Nevertheless, the analysis of linkage disequilibrium (LD) between CNVs and SNPs indicates that distinct copy numbers often sit on their own background haplotypes.

**Results:**

We propose new computational models for inferring chromosome-specific copy numbers by distinguishing background haplotypes of each copy number. The formulated problems are shown to be NP-hard and approximation/heuristic algorithms are developed. Simulation indicates that our method is accurate and outperforms the existing approach. By testing the program in 60 parent-offspring trios, the inferred chromosome-specific copy numbers are highly consistent with the law of Mendelian inheritance. The distributions of copy numbers at chromosomal level are provided for 270 individuals in three HapMap panels.

**Conclusions:**

The estimation of chromosome-specific copy numbers using microarray or sequencing platforms was often confounded by a number of factors. This study showed that the integration of background haplotypes is able to improve the accuracies of copy number estimation at chromosome level, especially for the CNVs having strong LD with SNPs in proximity.

## Background

Genetic variations exist in many forms in the human genome. Large structural variations such as deletions and duplications are quite common in the human populations, which encompass more base pairs than single nucleotide polymorphisms (SNPs). Among various types of structural variations, copy number variations (CNVs) often occupy regulatory regions of genes and greatly influence phenotypic traits and disease susceptibility [1]. CNV is defined as a DNA segment with length more than 1 kb and observed with various numbers of copies in the population. A number of CNVs have been known to highly associate with several complex diseases such as HIV infection, autoimmunity, autism, Parkinson's, Alzheimer's and Crohn's disease [2-6].

The advance of high-throughput array platforms and sequencing technologies enables fast and cost-effective scan of CNVs in genome-wide scale [7]. Using array Comparative Genomic Hybridization (aCGH) and next-generation sequencing, a large number of CNVs have been identified in human and other primates [8-12]. In practice, because our human genome is a diploid, most sequencing platforms often report total copy numbers of one individual instead of chromosome-specific copy numbers presented on each of the two homologous chromosomes. For example, suppose there are two diplotype configurations at one CNV locus: 1/1 represents one copy at each of the two chromosomes, and 0/2 indicates a deletion at one chromosome and a duplication at the other. The total copy numbers of these configurations are both experimentally obtained as two, although the underlying mechanisms generating these two configurations are different. Nevertheless, determination of chromosome-specific copy numbers is important in the analysis of population genetics and disease association studies. For instance, the power of detecting positive selection and accuracy of measuring Linkage Disequilibrium (LD) between SNPs and CNVs can be improved through direct use of chromosome-specific copy numbers [13-15]. Moreover, identification of the chromosome-specific copy numbers can even shed light on the age of each CNV [1].

Recently, an expectation maximization (EM) algorithm was developed to estimate frequencies of chromosome-specific copy numbers under the assumption of Hardy-Weinberg equilibrium (HWE) [16]. In reality, the observed allele frequencies do not completely satisfy HWE, because each copy number allele may be sampled more or less in different sequencing projects. In a few occasions, HWE may be even deviated due to directional selection, assortative mating, or migration [17]. Using B allele frequencies (BAF) and log R ratios (LLR) provided by SNP array platforms, a hidden Markov model (HMM) was designed for inferring chromosome-specific copy numbers within a parent-offspring family [18]. In addition, information of allelic-specific copies at each SNP locus (e.g., AAABB) have been also used to indirectly infer chromosome-specific copy numbers [19]. However, BAF, LLR and allelic-specific copies are not always available in each sequencing platform. For example, in next-generation sequencing (e.g., SOLid and Illumina), SNPs and CNVs called at these platforms (e.g., Bioscope and SAMTools) do not provide such information. Moreover, the accuracy of allelic-specific copies is often decreased for higher copies and is worse than that of total copy numbers due to cross-hybridization [20,21]. Although traditional haplotype phasing programs (e.g., fastPHASE) may be used for inferring copy number by encoding bi-allelic CNVs into SNP genotypes, this approach is inadequate to infer multi-allelic CNVs [13,19].

To date, the analysis of LD structure in human genome indicated that many CNVs have strong LD with SNPs in proximity, probably owing to uneven distribution of recombination hot/cold spots or genetic hitchhiking [22-25]. Moreover, a number of CNVs have been shown to be taggable using alleles at flanking SNPs [3]. The LD structure between CNVs and SNPs implies different chromosome-specific copy numbers often sit at their own background haplotypes, which can be viewed as fingerprints of each copy number. As a consequence, chromosome-specific copy numbers of each individual are inferable by careful analysis of background haplotypes around each CNV. In recent years, several large-scale sequencing projects have constructed complete haplotype and CNV databases across major human populations (e.g., HapMap [26]). Integration of these databases may gain insight into the distribution of chromosome-specific copy numbers in human populations.

In this study, we develop new computational models and combinatorial algorithms for inferring chromosome-specific copy numbers by distinguishing background haplotypes of each copy number. Two optimization problems are formulated, shown to be NP-hard, and solved by approximation or heuristic algorithms. Simulation indicates our method is very accurate and is able to outperform existing approach. By testing the program separately for each individual within 60 parent-offspring trios, the inferred chromosome-specific copy numbers are highly consistent with the law of Mendelian inheritance. The distribution of chromosome-specific copy numbers across three human populations indicate that one copy is the major allele as expected, and zero copy (deletion) alleles are much frequent than high copy (duplication) alleles.

## Methods

The haplotypes of 270 individuals are downloaded from the Phase II of international HapMap project [26]. For the input of unphased genotypes, the haplotypes were inferred via the PHASE [27] program, which was used by the HapMap project. For high-throughput sequencing data, a number of haplotype assembly tools can also be used to infer the haplotypes [28]. The total copy numbers of 1,319 CNVs typing on the same individuals are retrieved from [9]. We extract SNPs within each CNV as well as SNPs at flanking regions in our study. We compared the SNP distance (i.e. number of SNPs) with the physical distance (e.g., 5 kb) for capturing the extent of LD and found that the LD is more sensitive to physical distance. The simulation results indicated that the accuracy of our algorithm is highest when including SNPs within one-fold extension of the physical size of each CNV (Additional file 1, Figure S1). Therefore, the released program will automatically checks the coordinates of CNVs and SNPs and captures SNPs within the one-fold extension regions into consideration.

**Figure 1:**
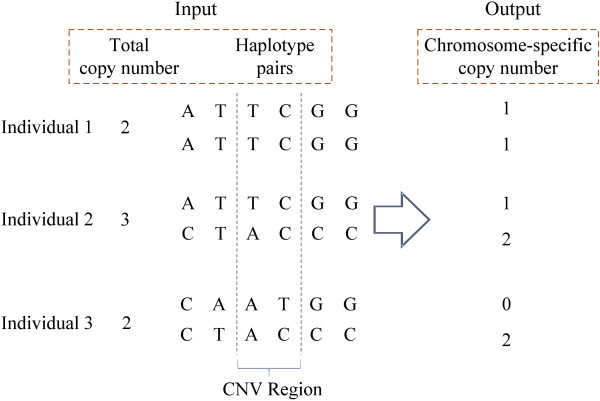
**Input and output examples**. An example of input and output of our program.

Given a set of haplotype pairs and the total copy number for each individual (Figure 1), the chromosome-specific copy number of each haplotype is determined by first solving a variant of Max-*k*-Cut problem, which aims to divide background haplotypes into *k *clusters. Then, a variant of Constraint Satisfaction Problem (CSP) is solved to assign chromosome-specific copy number to each cluster. Finally, these two procedures are repeated for all possible *k *in order to determine the best solution.

### Haplotypes Clustering via Solving Constrained Max-*k*-Cut Problem

**Figure 2:**
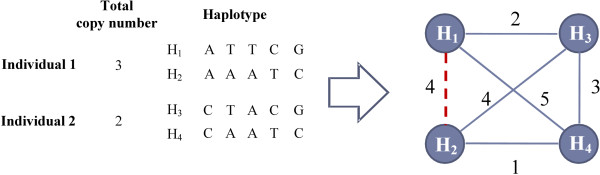
**Formulation of CNVs/haplotypes into a weighted graph**. Formulation of CNVs/haplotypes into a weighted graph with hard and soft edges. The dotted line denotes the hard edge, because the total copy of *H*_1 _and *H*_2 _is an odd number.

Through analysis of LD between SNPs and CNVs, the copy numbers on a CNV are shown to have strong LD with alleles at flanking SNPs [13,23,24]. The LD structure implies different chromosome-specific copy numbers tend to sit at their own background haplotypes. We first group haplotypes spanning across each CNV into *k *clusters (for all possible *k*) based on their pairwise hamming distance and total copy numbers. Note that odd total copy number implies the underlying two chromosome-specific numbers should be different (e.g., 3 = 0 + 3 or 1 + 2). Haplotypes clustered into the same set may represent haplotype background for the same chromosome-specific copy number. The input total copy numbers and haplotypes are formulated into a weighted graph described as following (see Figure 2):

(1) Each haplotype is transformed into a vertex.

(2) The weight of edge between two vertices is the hamming distance between two haplotypes. Note that the same haplotype from different individuals are formulated as multiple vertices with zero distance.

(3) For the haplotype pair with odd total copy number (e.g., *H*_1 _and *H*_2_), the edge between them is called *hard edge*.

(4) For the other haplotype pairs, the edges between them are called *soft edges*.

Given the above weighted graph with hard and soft edges, these haplotypes are grouped into *k *clusters by solving a variant of Max-*k*-Cut problem (called constrained Max-*k*-Cut). A formal definition of the constrained Max-*k*-Cut problem is given below.

### Problem: Constrained Max-*k*-Cut

Given an undirected weighted graph *G *= (*V, E*) in which some edges in *E *are hard and the others are soft, the constrained Max-*k*-Cut problem aims to find a partition of vertices in *V *into *k *sets (*X*_1_, *X*_2_, ..., *X_k_*) such that the total weight of soft edges across different sets (called cut) is maximized, requiring all hard edges must be on the cut.

The original Max-*k*-Cut is known to be NP-hard [29,30], which is a special case of this problem when all edges are soft. Therefore, the problem of constrained Max-*k*-Cut is also NP-hard. In order to efficiently solve the constrained Max-*k*-Cut problem, we develop a greedy approximation algorithm which explores larger solution space by randomizing non-deterministic steps. The core procedure of this algorithm is given below (Additional file 2, Figure S2).

### Algorithm for Constrained Max-*k*-Cut

(1) Randomly pick *k *different vertices as initial elements for each *k *set (*X*_1_, *X*_2_, ..., *X_k_*).

(2) Without violating the constraint of hard edge, randomly pick a remaining vertex and assign it into the set which maximizes the total weight of soft edges across different sets. This step is repeated until all vertices are assigned.

Note that the above procedure involves non-deterministic parts in both steps (i.e., initial *k *vertices and the order of picking the next vertex). Therefore, this procedure is repeated ten times to explore larger solution space by trying different initial *k *vertices in step 1 and different order in step 2. The best solution among all trials is outputted as the final solution. The number of repeated iterations is usually a tradeoff between accuracy and efficiency. Nevertheless, we found that the randomized approaches on top of the greedy framework requires only few iterations (Additional file 3, Figure S3). Thus, the implemented can run fast in practice. The following theorem implies that the solution found by this algorithm is quite close to the optimal solution.

**Theorem 1**. *The algorithm for constrained Max-k-Cut is a *(*k ***- **2)/(*k ***- **1)*-approximation algorithm for k *> 2.

*Proof*. Without loss of generality, let the order of picking vertices be *v*_1_, *v*_2_, ⋯, *v_n_*. Let *W *denote the total weight of all edges in *G *and

then

Let *X_j _*be the *j*-th set of partitioned vertices and

where 1 ≤ *m *≤ *i *- 1 , 1 ≤ *j *≤ *k *. Then

Suppose Cut_*i*1 _≥ Cut_*i*2 _≥ ⋯ ≥ Cut*_ik _*and *v_i _*cannot be put into set *X*_1 _due to the hard-edge constraint. Then *v_i _*can be put into set *X*_2 _by the algorithm instead, because the hard edge only appears between haplotype pairs of the same individual. The relation between Cut*_ij _*and *W_i _*can be computed as following:

Therefore,

Let *C *denote the solution from the greedy algorithm and C* be the optimal solution, then

Since W ≥ C*,

### Copy Number Assignment via Solving Constraint Satisfaction Problem

**Figure 3:**
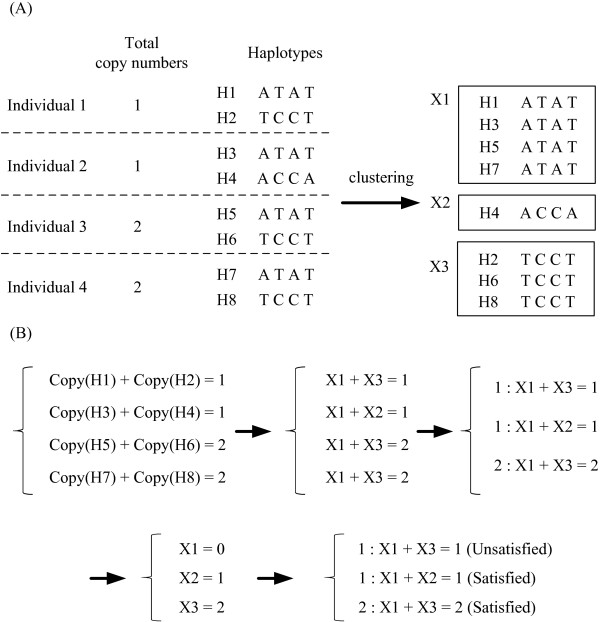
**Haplotype clustering and copy number assignment**. Examples of (A) haplotype clustering and (B) Assignment of chromosome-specific copy number.

After clustering haplotypes into *k *sets (*X*_1_, *X*_2_, ..., *X_k_*), we then assign *k *different integers to each set, which correspond to *k *distinct chromosome-specific copy numbers. For each individual, summation of chromosome-specific copy numbers of each haplotype pair should be equal to his/her total copy number. which can be written as the following constraint:(1)

where Copy(H_*i*1_) and Copy(H_*i*2_) are chromosome-specific copy numbers for the *i*-th individual, and Total(*i*) is his/her total copy number. For the example shown in Figure 3(A), Copy(H_5_)+Copy(H_6_) = 2 for individual 3. Note that because all haplotypes have been clustered into the same or different sets, eq (1) can be rewritten into the following constraint using their set variables *X_i_*:(2)

where *X_a _*and *X_b _*denote the sets of these two haplotypes after clustering (e.g., *X*_1 _+ *X*_3 _= 2 for individual 3). For each individual, a set of constraints with two variables similar to eq (2) can be generated by repeating the above formulation (see Figure 3(B)). By assigning distinct integer numbers to these set variables *X_i_*, chromosome-specific copy number of each haplotype can then be determined. Theoretically, all constraints should be satisfied after the assignment, but practically, not all constraints can be satisfied, because some ambiguous haplotypes may not be the true background of the copy number. In order to satisfy as many constraints as possible, chromosome-specific copy numbers are assigned to each set *X_i _*by solving a variant of the constraint satisfaction problem (termed Unique Max-2-CSP). Given a set of two-variable constraints over *n *variables (*X*_1_, *X*_2_*,..., X_n_*), the Unique Max-2-CSP problem asks for *k *unique (distinct) integers assigned to each variable which satisfied maximum number of constraints.

### Problem: Unique Max-2-CSP

Given a set of variables *X *= {*X*_1_, *X*_2_, ..., *X_n_*}, a set of finite integer domains *D *= {0, 1, ..., *d*}, where *d *≥ *n - *1, and a set of two-variable constraints *C *= {*C*_1_, *C*_2_, ..., *C_m_*} with the following form:(3)

where *T_l _*is a non-negative integer. The Unique Max-2-CSP asks for an assignment of *n *distinct integers in *D *to *X*_1_, *X*_2_, ..., *X_n _*that maximizes the total number of satisfied constraints in *C*.

We first prove a problem called binary Max-2-CSP is NP-hard, in which the integer domain *D *is restricted to {0, 1}, and values assigned to different variables in *X *are allowed to be identical (e.g., *X*_1 _*= X*_2 _*= *1).

Then, the unique Max-2-CSP problem is shown to be NP-hard by reduction from binary Max-2-CSP. The details of these proofs can be found in Additional file 4, Supplementary Material.

**Theorem 2**. *Unique Max-2-CSP is NP-hard*.

In order to solve unique Max-2-CSP more efficiently, we developed a greedy heuristic algorithm which also explores larger solution space by randomizing non-deterministic steps. Let *n *be the number of individuals and *c_max _*be the maximum possible copy number.

### Algorithm for Unique Max-2-CSP

**Figure 4:**
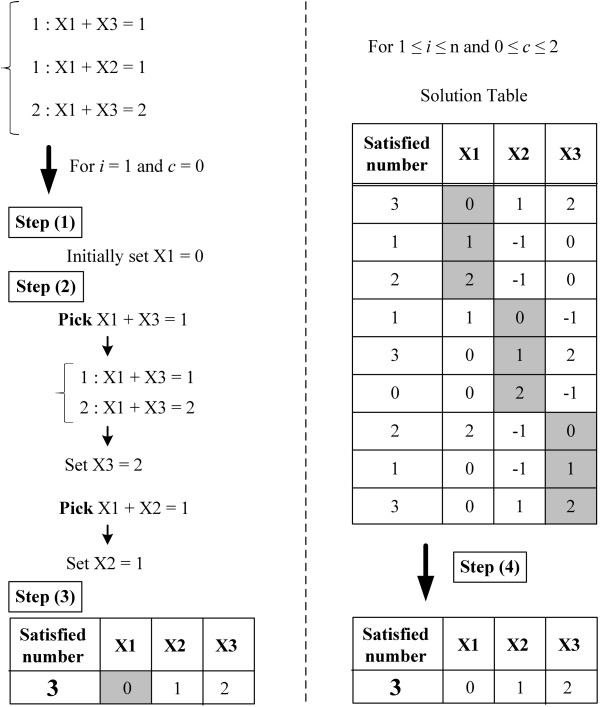
**The algorithm of assigning chromosome-specific copy number**. An example of the algorithm for assigning copy number by solving unique Max-2-CSP.

For 1 ≤ *i *≤ *n*, 0 ≤ *c *≤ *c_max_*, do step (1) to step (3) (see Figure 4).

(1) Initially set *X_i _*= *c*.

(2) Randomly pick a constraint {*N *: *X_a _*+ *X_b _*= *T *} in which only *X_a _*(or *X_b_*) is assigned, where *N *is the number of the constraint. If *X_a _*(or *X_b _*) = *D*, and there are *m *types of constraints with *X_a _*+ *X_b _*as following: {*N_j _*: *X_a _*+ *X_b _*= *T_j _*}, where 1 ≤ *j *≤ *m*, and *N*_1 _is maximum in *N_j_*, assign *X_b _*(or *X_a _*) = *T*_1 _- *D*. Repeat this step until there is no constraint in which only one variable is assigned.

(3) Compute the number of satisfied constraints with respect to *X_i _*= *c*.

Ideally, once the value of the initial variable *X_i _*is assigned (e.g., *X*_1 _= 0), the values of other associated variables can be indirectly determined (e.g., *X*_1 _+ *X*_2 _= 1 or *X*_1 _+ *X*_3 _= 2). However, there could be some conflicting constraints existed (e.g., *X*_2 _+ *X*_3 _= 3). Therefore, the possible values of all variables *X_i _*are dependent on the order of assignment (e.g., *X*_1 _= 0 first, *X*_2 _= 1 second, then ...). In reality, there are more variables and the dependency/conflict relations are more complicated. Consequently, we repeat the above procedure ten times to explore different orders of assignments by randomly prioritizing distinct constraints to be satisfied in different rounds. The best solution among all iterations is recorded into the corresponding row in the solution table. Note that some variables may have no assignment due to conflicts with previously assigned variables and hence are recorded as -1.

After the above procedure is iterated over possible initial values of all variables, a solution table will be created. Each row stands for one assignment corresponding to the initial value of some variable. Note that although each row represents a set of possible assignment, the assignment may not satisfy all variables due to the lack of dependency with other variables (e.g., *X*_4 _may not be reachable from *X*_1_). Therefore, we do step (4) iteratively using a greedy approach.

(4) Select a row which is not chosen in the solution table with maximum number of satisfied constraints. Repeat this row selection until no further constraints can be satisfied. Note that the variables assigned in one iteration cannot violate the assignment in previous iteration.

Finally, the union of assignments selected by this greedy algorithm is outputted as the solution.

### Iteration and Adjustment

**Figure 5:**
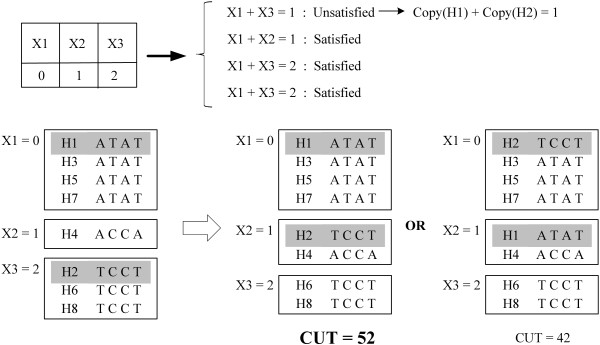
**Adjustment for the individuals with unsatisfied constraints**. An example of adjusting clustering result for the individuals with unsatisfied constraint. The first constraint is unsatisfied for X1 = 0, X2 = 1 and X3 = 2. Adjust the clustering result by putting H1 in X1 and putting H2 in X2 for satisfying the first constraint and maximizing the cut size.

The previous two procedures (haplotype clustering and copy number assignment) are repeated for all possible numbers of clusters *k*, because the best setting of *k *can not be known in advance. We try all possible *k *from two to maximum possible number. For example, if a CNV have total copy number 2, 3, 4 in populations, the maximum possible *k *is 5 since all possible chromosome-specific copy numbers range from 0 to 4. We choose the best *k *with maximum number of satisfied constraints in unique Max-2-CSP. In practice, the constraints of some individuals may be still unsatisfied after these iterations, because the ambiguous haplotypes, which are not the true background of underlying copy number, may confuse the haplotype clustering. Consequently, we adjust the clustering results for these unsatisfied individuals using the following randomized approach (see Figure 5 for an example):

(1) Randomly pick an individual with haplotypes violating the constraint and enumerate all possible assignments for these two haplotypes such that the constraint can be satisfied.

(2) For each possible assignment, evaluate the new cut value in the Max-k-Cut problem and choose the assignment with maximum cut among all possibilities.

(4) Repeat step (1) and step (2) for the remaining unsatisfied individuals until all of them are satisfied.

Because the order of individuals processed is non-deterministic, we also repeat above procedure ten times and output the best solution among them.

### Simulation

The simulation of LD and HWE generated two series of copy numbers and SNP genotypes from 16 individuals. The haplotype phases are inferred via PHASE [27]. The first series of data sets simulate diplotype configurations completely match HWE (*P *= 1.0, Chi-square). The flanking SNPs are simulated starting from perfect LD (average *r*^2 ^= 1.0). Subsequently, the remaining data sets of lower LD are constructed by flipping SNP alleles at random. The second set of experiments simulated an imperfect HWE data sets by adding/deleting some copy number alleles from the HWE data sets, which aims to slightly deviate from the expected HWE frequency (*P *= 0.98). The remaining data sets of LD decay are generated in a similar way.

The simulation using copy number on X Chromosomes is also adopted. Because there is only one X chromosome in each male, the total copy number obtained on X chromosome directly represents the chromosome-specific copy number [9]. We use CNVs and haplotypes in X chromosomes of males from the HapMap project during simulation. The total copy numbers at one CNV is simulated by randomly pairing two copy numbers on two different X chromosomes.

In order to compare the accuracy of our algorithm and CNVphaser [1,16], which outputs posterior probabilities of each copy number, we parsed the output files of CNVPhaser and picked up the diplotype configuration with highest probability for each individual. The accuracy of inferred copy number configurations is defined as following:

where *C_correct _*is the number of correctly inferred copy number configurations and *C_total _*is the total number of configurations.

## Results

The proposed algorithms have been implemented as a program called CSCNPhaser, which is available at http://www.cs.ccu.edu.tw/~ythuang/Tool/CSCNPhaser/. We retrieved haplotypes of 270 individuals from Phase II of the International HapMap Project [26]. These individuals include 30 trios from the Utah, USA region (CEU); 30 trios from the Yoruba in Ibadan, Nigeria (YRI); 45 unrelated Japanese individuals from Tokyo, Japan (JPT); and 45 unrelated Han Chinese individuals from Beijing, China (CHB). In addition, total copy numbers at 1,319 CNVs typing on the same 270 individuals are downloaded from [9]. We consider haplotypes within the CNV as well as haplotypes at flanking regions, whereas the best length of extended haplotypes is determined by simulation (see Method).

### Simulation on LD Decay and HWE

**Figure 6:**
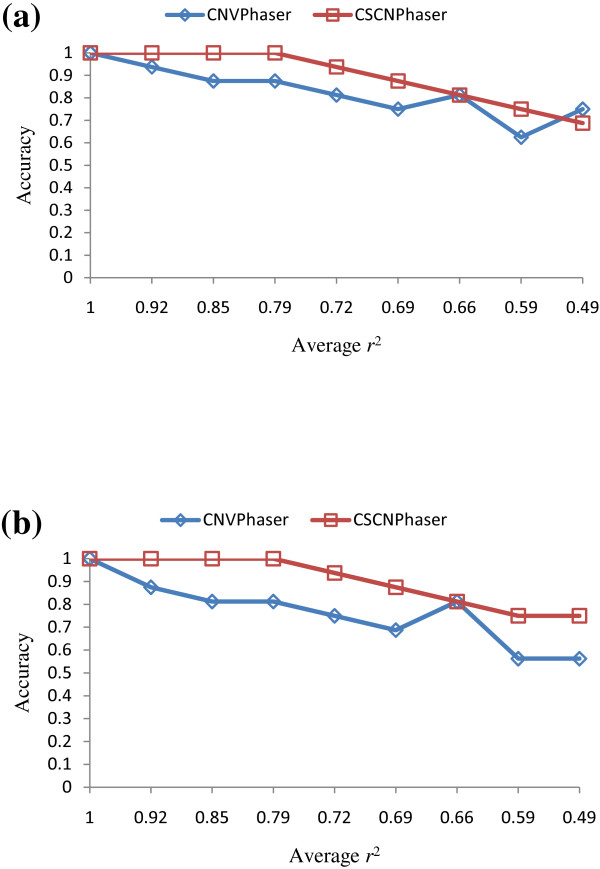
**Accuracies of inferred chromosome-specific copy number**. (A) The average accuracy of CNVphaser and CSCNPhaser under complete HWE and various LD. (B) The average accuracies of CNVphaser and CSCNPhaser under near HWE and various LD.

We compared the CNVPhaser [1,16] and our program CSCNPhaser over two series of data sets with respect to different LD and HWE (see Method). Although the copy numbers in both experiments almost match the ideal HWE, the slight deviation from HWE is shown by the *P *values using the Chi-square test. The first set of experiments simulated complete HWE, in which the copy alleles in all data sets completely follow the expected frequency (*P *= 1.0). The flanking SNP alleles are randomly flipped to decay the LD. Figure 6(A) plots the accuracies of CSCNPhaser and CNVPhaser at different degrees of LD under complete HWE. Because CSCNPhaser is designed based on the LD of background haplotypes, the accuracy is decreasing as the background haplotypes are less LD-informative. Unexpectedly, we found the accuracy of CNVPhaser also deteriorates as LD decays. This is because CNVPhaser estimated the combined frequencies of the entire haplotype and copy number to match HWE, which implicitly captured LD in a light way. CSCNPhaser outperforms CNVPhaser as the background haplotypes are more LD-informative, and both accuracies are worse as haplotypes are less informative.

The second set of experiments simulated an imperfect HWE data sets by adding/deleting a few copy number alleles to slightly deviate from the expected HWE frequency (*P *= 0.98). Note that the entire allele frequency spectrum is still close to that of HWE. Figure 6(B) plots the accuracies of CSCNPhaser and CNVPhaser at different degrees of LD. In high LD, both programs can still achieve high accuracies.

Although the major trends are similar to previous experiments, CNVPhaser is slightly worse than previous experiment compared with our method, implying it is more sensitive to HWE deviation.

### Consistency with Mendelian Inheritance

**Figure 7:**
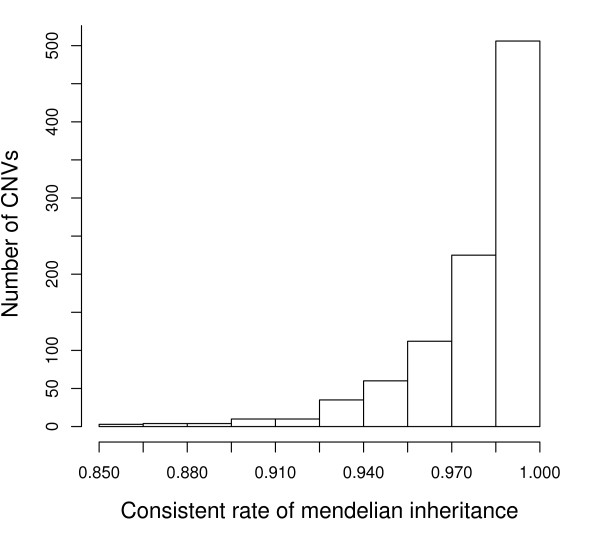
**Mendelian consistency in parent-offspring trios**. Consistent rates of Mendelian inheritance of inferred chromosome-specific copy numbers in 60 parent-offspring trios.

The developed program is further applied on 1,292 CNVs on autosomal chromosomes typing over 270 HapMap individuals from [9]. We discarded CNVs with less than 10 SNPs, because they are less informative about LD. There are 969 CNVs used in following experiments. The copy numbers observed among normal individuals should be overwhelmingly inherited from their parents. By running our program separately for each individual within 60 parent-offspring trios (CEU and YRI panels), correctness of our method can be justified by checking the Mendelian consistent rate of inferred chromosome-specific copy numbers within trios [9]. More than 97 percent of CNVs have Mendelian consistent rate larger than 0.9 (see Figure 7). These results indicate that majority of copy numbers inferred by our method is under expectation from the law of Mendelian inheritance. The remaining few CNVs might imply novel deletions/duplications or translocation-mediated CNVs [31,32].

### Distributions of Copy Number Configurations in Human Populations

**Figure 8:**
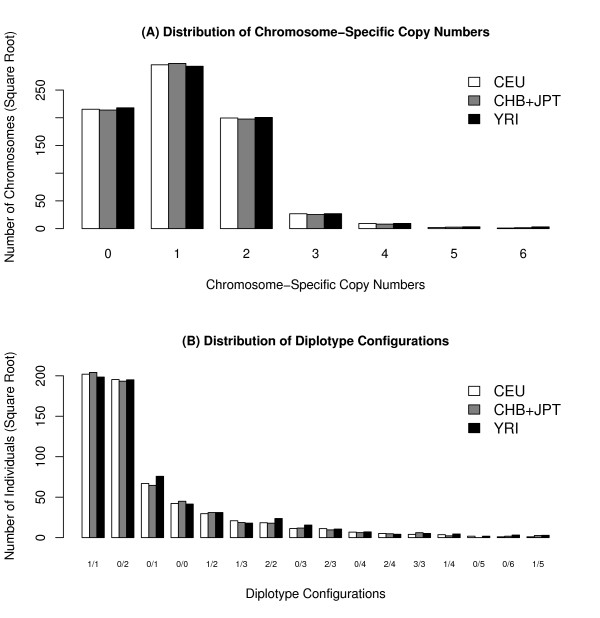
**Distributions of chromosome-specific copy numbers and diploid copy number configurations**. Distributions of chromosome-specific copy numbers and diploid copy number configurations. (A) Distribution of chromosome-specific copy numbers in three HapMap panels. (B) Distribution of diploid configurations in three HapMap panels.

The chromosome-specific copy numbers of 270 individuals in CEU, YRI, and CHB+JBP HapMap panels are inferred by our program in order to investigate the distributions of haplopid and diploid configurations in human populations. Figure 8(A) plots the haploid distribution of chromosome-specific copy numbers inferred by our program. Our results indicate that one copy on each chromosome is the major allele in the population as expected. Zero copy (deletion) is the second frequent allele compared with two copies (duplication). Frequencies of higher chromosome-specific copy numbers are relatively lower. This is not unexpected because multiple duplication events at the same CNV locus are relatively less common.

**Figure 9:**
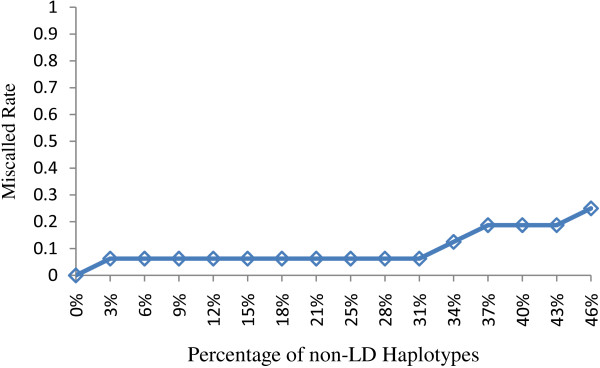
**The miscalled rates of 1/1 configurations to 0/2 configurations**. The miscalled rates of 1/1 configurations to 0/2 configurations with respect to the percentage of non-informative haplotypes.

In the distribution of diploid configurations (Figure 8(B)), 1/1 configurations are the most frequent form as expected. We observed 0/2 configurations (deletion+duplication) is the second frequent one. This phenomenon may be explained by the fact that 1/1 and 0/2 configurations contribute equally to gene copy balance in humans. In order to assess the miscalled rates of 1/1 into 0/2 configurations, we conducted a series of simulation experiments of only 1/1 diplotype configurations (i.e., no CNV). Because 1/1 configuration is miscalled to 0/2 by CSCNPhaser only when the background haplotypes are not LD-informative, we investigated the miscalled rates of data sets starting from LD-informative haplotypes down to low-LD ones. Specifically, the haplotypes are one-by-one replaced with non-informative haplotypes. Figure 9 plots the miscalled rates with respect to the percentage of replaced haplotypes. When the majority of haplotypes are LD-informative (>60%), the miscalled rate is low (~0.06). As more haplotypes are replaced with non-informative ones, the miscalled rate goes up as expected.

On the other hand, 0/1 and 0/0 configurations (hemizygous and homozygous deletions) are more frequent than the remaining duplication forms, probably due to the low frequencies of high-copy alleles. Although these distributions are consistent across three HapMap panels, these results could be still biased due to low-LD background of recurrent or translocation-mediated CNVs. Therefore, these distributions are for reference only, which require experimental validations before further interpretation.

### Capability and Efficiency

**Figure 10:**
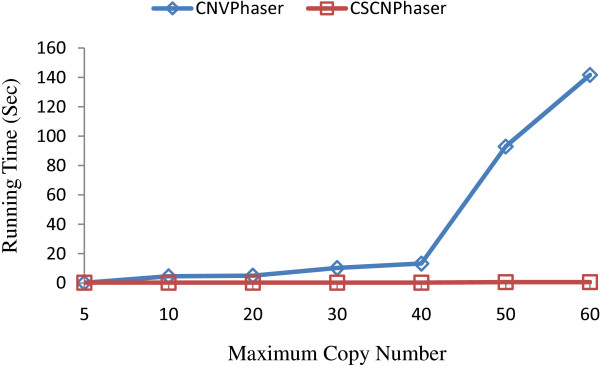
**Comparison of capacity and efficiency**. The running time of CNVPhaser and CSCNPhaser for processing data sets with different maximum copy numbers.

Although the maximum copy number in the population is still not clear, it is worth of interest to know the capability and efficiency of both programs for processing data sets with large copy numbers. Figure 10 plots the average running time of CNVPhase and CSCNPhaser over a range of maximum copy numbers. Both programs are able to accept input of up to 60 copies. The differences are the running time and memory usage. CNVPhaser requires longer time (>1 min) and more memory (>1GB) for >50 copy numbers, whereas CSCNPhaser is very fast (within seconds) and does not consume much memory.

## Discussion

### Strength and Weakness of LD-based Inference

CNVPhaser was developed by estimating allele frequencies using HWE, while our CSCNPhaser investigated the haplotype background of each copy number. Although not explicitly stated, we observed CNVPhaser implicitly capture background haplotypes in a light way, because the frequencies are estimated over the entire copy number/haplotype combinations. Therefore, its accuracy also decreases as LD decays. For CNVs having high LD with flanking SNPs, our program performs better than CNVPhaser. In low LD regions with only 1/1 configurations, we observed that CSCNPhaser may miscall them as 0/2 configurations. On the other hand, in data sets with mixed configurations (i.e., 1/1 and 0/2), we observed that the miscalled rate is lower, because these data sets contain haplotypes LD-informative of 0/2 configuration, which are used for better distinguishing 1/1 from 0/2 configurations in our algorithm. Most common, diallelic CNVs have been found to have strong LD with flanking SNPs, and most low-frequency CNVs even segregated on specific haplotype background [9]. Therefore, we anticipate it is useful to look at the haplotype background for inferring copy number of most CNVs.

It should be noted that the LD-based approach is not suitable for recurrent CNVs or translocation-mediated CNVs, in which their background haplotypes are less informative of the copy number. In fact, our simulation on X-chromosome CNVs found two possible recurrent CNVs with lower accuracies compared with other ordinary CNVs (Tables 1 and 2). Nevertheless, the CNVPhaser and our program can work in a hybrid way to overcome the limitation. The LD of SNPs across the CNV can be computed first. If the LD is low (i.e., recurrent CNVs), it might be a clue for not looking into the haplotypes for copy number inference. That is, we can run the CNVPhaser but exclude SNP genotypes for pure HWE frequency estimation. As for other CNVs with LD-information haplotypes, our program can be used to achieve higher accuracies. With the release of next generation sequencing platforms, SNPs and CNVs are often collectively called in each sequencing project. And the accuracies of inferring these CNVs can be improved by further looking into the LD background of each copy number.

**Table 1:** Accuracies on X chromosome Simulation.

CNV IDs	Exp. 1	Exp. 2	Exp. 3	Exp. 4	Exp. 5
16 CNVs	0.788272	0.828395	0.869445	0.917284	0.953704

CNV2621	0.585185	0.618519	0.611111	0.666667	0.685185

CNV2682	0.314815	0.222222	0.359259	0.292593	0.285185

The average accuracies of 16 ordinary CNVs compared with those of two recurrent CNVs.

**Table 2:** Copy numbers of the 18 CNVs on X chromosomes.

**CNVid**	**Copy Number**	**CNVid**	**Copy Number**
	
2593	0,1	2654	0,1
	
2603	0,1	2659	0,1
	
2604	0,1	2675	0,1
	
2619	0,1	2678	0,1
	
**2621**	**0,1,2**	**2682**	**1,2,3**
	
2627	0,1	2694	0,1
	
2636	1,2	2704	0,1
	
2639	1,2	2706	0,1
	
2648	0,1	2707	1,2

The copy numbers at 18 CNVs on X chromosomes in males. Two possible recurrent CNVs are highlighted.

### Strategies for Solving Constrained Max-*k*-Cut

The original Max-*k*-Cut problem can be solved by a randomized algorithm which randomly partition all vertices into *k *sets. In addition, it can be also solved by a deterministic greedy algorithm which iteratively assigns one vertex into the set that maximizes the cut size. In fact, both algorithms return a good approximate solution within a factor of  of the optimal solution, and the semidefinite-programming (SDP) relaxation can achieve a better approximation bound [30]. Therefore, it is natural to consider the three strategies for solving the constrained version. Although not presented in this paper, the random partition method was ever considered but later withdrawn due to the bad accuracies in all experiments. It is because the random partition method simply guess the solution, which can't work in practice although the approximation ratio is theoretically good. The SDP strategy is theoretically sound but the running time is slow in our previous study [33], and the SDP implementation is complex so that the program is often not easily portable to all platforms. On the other hand, the greedy algorithm (with randomization enhancement) can achieve high accuracies and run very fast in all experiments. As a consequence, the greedy solution is taken in order to perform genome-wide experiments with high accuracies and within reasonable period of time.

### Integration of Greedy and Randomized Approaches

Theoretically, the two optimization problems (Max-*k*-Cut and Max-2-CSP) can be both solved by a deterministic greedy approach or a pure randomized approach (e.g., random partition for the *k*-cut problem). The greedy approach is simple and fast. However, the solution found is often only theoretically sound but not comparable with other heuristic methods in practice. This is due to the fact that the ordinary greedy approach tends to find local optimum solution instead of global optimum solution. On the other hand, the pure randomized (blind-search) approaches do not have the tendency of finding local optimum solution but requires numerous iterations for finding a good solution. Therefore, when solving both algorithms, we used the greedy algorithm as a framework and randomized the non-deterministic steps for searching better solutions. The results showed that the iterations required of this hybrid approach are far less than those of pure randomized approaches, while obtaining better solutions than an ordinary greedy algorithm.

## Conclusion

In this study, we developed new computational models and combinatorial algorithms for inferring chromosome-specific copy numbers by distinguishing their haplotype background. Simulation showed that our method is accurate and outperformed existing method as the background haplotypes are LD-informative of the copy numbers. The inferred copy numbers are consistent with Mendelian inheritance for 97% of CNVs within parent-offspring trios. The inference of copy numbers in microarray and sequencing platforms are often confounded by a number of different factors. This study showed that integration of haplotypes into copy number estimation is able to improve the accuracies, especially for those CNVs having strong LD with SNPs.

## Authors' contributions

YTH formulated the two optimization problems. YTH and and MHW designed and analyzed the proposed algorithms. YTH and MHW conducted experiments and wrote the manuscript. Both authors read and approved the final manuscript.

## Supplementary Material

Additional file 1 **Supplementary Figure S1**. Simulation of extended regions to the left or right of a CNV. The results indicate accuracy is highest when the extension is equal to the size of CNV. Click here for file

Additional file 2 **Supplementary Figure S2**. An example of the algorithm for solving the constrained Max-k-Cut problem. Click here for file

Additional file 3 **Supplementary Figure S3**. The average accuracies of CSCNPhaser with respect to different iterations used. Click here for file

Additional file 4 **Supplementary Material**. Other Methods and proofs of theorems in this paper. Click here for file
