# Supplementary material for: Inference of chromosome-specific copy numbers using population haplotypes
Source: BMC Bioinformatics. 2011 May 24;12:194. doi: 10.1186/1471-2105-12-194 (PMC3128032; doi:10.1186/1471-2105-12-194)
Supplement: Additional file 4 — Supplementary Material. Other Methods and proofs of theorems in this paper. [file 1471-2105-12-194-S4.PDF]

Supplementary Material: Inference of Chromosome-Specific Copy Numbers Using Population Haplotypes

Yao-Ting Huang[1]*and Min-Han Wu[1]
[1]Department of Computer Science and Information Engineering
National Chung Cheng University, Chiayi, Taiwan
ythuang@cs.ccu.edu.tw

1 Hardness of Unique Max-2-CSP

Problem: Unique Max-2-CSP. Given a set of variables $X = \{X_1, X_2, ..., X_n\}$, a set of finite integer domains $D = \{0, 1, ..., d\}$, where $d \geq n - 1$, and a set of two-variable constraints $C = \{C_1, C_2, ..., C_m\}$ with the following form:

$$C_l : X_i + X_j = T_l, \text{ for all } 1 \leq l \leq m, \tag{1}$$

where T_l is a non-negative integer. The Unique Max-2-CSP asks for an assignment of n distinct integers in D to $X_1, X_2, ..., X_n$ that maximizes the total number of satisfied constraints in C.

We first prove a problem called binary Max-2-CSP is NP-hard, in which the integer domain D is restricted to $\{0, 1\}$, and values assigned to different variables in X are allowed to be identical (e.g., $X_1 = X_2 = 1$). Then, the unique Max-2-CSP problem is shown to be NP-hard by reduction from binary Max-2-CSP.

Problem: Binary Max-2-CSP. Given a set of variables $X = \{X_1, X_2, ..., X_n\}$, a set of binary integers $D = \{0, 1\}$, and a set of two-variable constraints $C = \{C_1, C_2, \cdots, C_m\}$ with the same form as eq (1). The binary Max-2-CSP problem asks for an assignment of n integers in D to $X_1, X_2, ..., X_n$ that maximizes the number of satisfied constraints.

The binary Max-2-CSP problem is shown to be NP-hard by reduction from a known NP-hard problem called Max-2-SAT.

Theorem 1. *Binary Max-2-CSP is NP-hard.*

Proof. We consider the decision versions of the binary Max-2-CSP and Max-2-SAT as follows:
Max-2-SAT: Given a conjunction normal form (CNF) over $\{X_1, X_2, ..., X_n\}$ boolean variables, where each clause contains exactly two literals, decide if there is a truth assignment satisfying at least k

*To whom correspondence should be addressed.

clauses.

Binary Max-2-CSP: Decide if there is an assignment in D to $X' = \{X'_1, X'_2, ..., X'_n\}$ satisfying at least k constraints.

Reduction: First, we convert the boolean literals in Max-2-SAT to integer variables in binary Max-2-CSP as following:

$$\begin{array}{llll} (1) & X_i & \rightarrow & X'_i \\ (2) & \bar{X}_i & \rightarrow & (1 - X'_i) \end{array}$$

Second, we reduce the clauses in Max-2-SAT to two constraints in binary Max-2-CSP as following:

$$(\mathrm{X}_i \vee \mathrm{X}_j) \rightarrow \begin{cases} X'_i + X'_j = 2 & \text{(a)} \\ X'_i + X'_j = 1 & \text{(b)} \end{cases}$$

Proof of reduction: There are k clauses satisfied in Max-2-SAT if and only if there are k constraints in binary Max-2-CSP are satisfied, since

(1) if $(X_i \vee X_j)$ is satisfied and X_i, X_j are both true, then $X'_i = 1$ and $X'_j = 1$, constraint (a) is satisfied.

(2) if $(X_i \vee X_j)$ is satisfied and exactly one of X_i, X_j is true, then either $X'_i = 1$ or $X'_j = 1$, constraint (b) is satisfied.

(3) if $(X_i \vee X_j)$ is unsatisfied (i.e., X_i and X_j are both false), then $X'_i = 0$ and $X'_j = 0$, and constraints (a) and (b) are unsatisfied.

On the other hand,

(1) if constraint (a) is satisfied (i.e., $X'_i = 1$ and $X'_j = 1$), then X_i and X_j are true and $(X_i \vee X_j)$ is satisfied.

(2) if constraint (b) is satisfied (i.e., $X'_i = 1$ or $X'_j = 1$), then X_i or X_j is true and $(X_i \vee X_j)$ is satisfied.

(3) if constraint (a) and constraint (b) are unsatisfied ($X'_i = 0$ and $X'_j = 0$), then X_i and X_j are false and $(X_i \vee X_j)$ is unsatisfied.

Since Max-2-SAT is NP-hard, binary Max-2-CSP is NP-hard. □

Next, we show unique Max-2-CSP is NP-hard by reduction from the binary Max-2-CSP problem.

Theorem 2. *Unique Max-2-CSP is NP-hard.*

Proof. We consider the decision versions of binary Max-2-CSP and Unique Max-2-CSP as follows:

Binary Max-2-CSP: Decide if there is an assignment of n integers in binary domain D to $X =$

$\{X_1, X_2, ..., X_n\}$ satisfying at least k constraints.

Unique Max-2-CSP: Decide if there is an assignment of m distinct integers in domain D' to $X' = \{X'_1, X'_2, ..., X'_m\}$ satisfying at least k constraints.

Reduction: First, each variable X_i in binary Max-2-CSP is converted to variables in Unique Max-2-CSP as following:

$$X_i \rightarrow \left\lfloor \frac{X'_i}{n} \right\rfloor$$

Second, constraints in binary Max-2-CSP are reduced according to the following rule:

$$X_i + X_j = T_l \rightarrow \left\lfloor \frac{X'_i}{n} \right\rfloor + \left\lfloor \frac{X'_j}{n} \right\rfloor = T_l$$

Third, we convert the domain $D = \{0, 1\}$ in binary Max-2-CSP to a larger domain $D' = \{0, 1, \cdots, 2n-1\}$ in unique Max-2-CSP, where n is the number of variables.

Proof of reduction: The reduction implicitly partitions the enlarged domain D' into two subdomains, where $X'_i \in \{0, 1, ..., n-1\}$ corresponding to $X_i = 0$, and $X'_i \in \{n, n+1, ..., 2n-1\}$ corresponding to $X_i = 1$. Note that the new domain D' allows all n variables assigned into the same subdomain in order to simulating identical assignments in binary Max-2-CSP. For any constraint in unique Max-2-CSP satisfied, X'_i or $X'_j \geq n$, implying the original constraint in binary Max-2-CSP can be also satisfied, and vice versa. Therefore, there are k constraints satisfied in binary Max-2-CSP if and only if there are k constraints satisfied in Unique Max-2-CSP.

Since binary Max-2-CSP is NP-hard, the unique Max-2-CSP problem is also NP-hard. □

2 Simulation on X Chromosome

The total copy number for each of the 18 CNVs on X chromosome is simulated by randomly pairing two copy numbers from two different X chromosomes. The random pairing is repeated five times to construct five data sets, and we computed the accuracies of CSCNPhaser running over the 18 CNVs. Among them, two CNVs are probably recurrent CNVs with more copy numbers and diverse haplotype background. We then compared the average accuracies of the remaining 16 ordinary CNVs with the two recurrent CNVs.
